# Softly empowering a prosocial expert in the family: lasting effects of a counter-misinformation intervention in an informational autocracy

**DOI:** 10.1038/s41598-024-61232-x

**Published:** 2024-05-23

**Authors:** Gábor Orosz, Laura Faragó, Benedek Paskuj, Zsófia Rakovics, Diane Sam-Mine, Gilles Audemard, Mouny Samy Modeliar, Péter Krekó

**Affiliations:** 1 ULR 7369 -URePSSS - Unité de Recherche Pluridisciplinaire Sport Santé Société, Sherpas, Univ. Lille, Univ. Littoral Côte d’Opale, Univ. Artois, Arras, France; 2 Institute of Psychology, ELTE Eötvös Loránd University, Budapest, Hungary; 3 Department of Psychology, University College London, London, UK; 4 Faculty of Social Sciences, Research Center for Computational Social Science, ELTE Eötvös Loránd University, Budapest, Hungary; 5 MTA–TK Lendület “Momentum” Digital Social Science Research Group for Social Stratification, HUN-REN Centre for Social Sciences, Budapest, Hungary; 6 CNRS, CRIL, Univ. Artois, Arras, France; 7 Political Capital Institute, Budapest, Hungary

**Keywords:** Family values, Eastern Europe, Misinformation, Prosocial motivations, Wise Intervention, Human behaviour, Social neuroscience

## Abstract

The present work is the first to comprehensively analyze the gravity of the misinformation problem in Hungary, where misinformation appears regularly in the pro-governmental, populist, and socially conservative mainstream media. In line with international data, using a Hungarian representative sample (Study 1, *N* = 991), we found that voters of the reigning populist, conservative party could hardly distinguish fake from real news. In Study 2, we demonstrated that a prosocial intervention of ~ 10 min (*N* = 801) helped young adult participants discern misinformation four weeks later compared to the control group without implementing any boosters. This effect was the most salient regarding pro-governmental conservative fake news content, leaving real news evaluations intact. Although the hypotheses of the present work were not preregistered, it appears that prosocial misinformation interventions might be promising attempts to counter misinformation in an informational autocracy in which the media is highly centralized. Despite using social motivations, it does not mean that long-term cognitive changes cannot occur. Future studies might explore exactly how these interventions can have an impact on the long-term cognitive processing of news content as well as their underlying neural structures.

## Introduction

In 2010, the Hungarian right-wing populist party Fidesz, led by Viktor Orbán, won a super-majority at the parliamentary elections and started dismantling democratic checks and balances^1^, solidifying their grip on power through multiple electoral cycles. Right-wing populist policies were implemented on a wide scale^2^, reflecting well a strong ideological stance of the regime, combining paternalist populism, illiberal conservatism, and civilizational ethnocentrism. This ideological mix is increasingly typical behind autocratization^3^. Furthermore, the regime gradually established an increasing media dominance, making possible the building of a parallel reality using state-sponsored dissemination of disinformation across the country—turning Hungary into an efficient informational autocracy^4^, with a declining level of freedom of the press^5^ (Freedom House, 2023). Based on the assessment of Mérték Institute^6^, 79% of the Hungarian media was controlled by the government or its proxies. Such concentration of media resources is unprecedented in the European Union and makes Hungary the country with the most centralized and ideologically controlled public space within the European Union. Few empirical studies have investigated the psychological ramifications of similar media landscapes on citizens. To our knowledge, even fewer have tried intervening to reduce disinformation susceptibility in such well-established political contexts^7^.

Hungary is an illiberal regime—or informational autocracy—characterized by systematic pro-governmental disinformation campaigns in the mainstream media^1,4,8–10^. Some experts categorize it as an informational autocracy (see e.g.,^4^) because of the increasing political influence in the media landscape, resulting in the shrinking of free and independent media^11^. The control of the media and the extensive use of disinformation unequivocally helped the Orbán-regime to keep power. For example, the Kremlin-inspired disinformation in the Hungarian public media about the Russian invasion of Ukraine could help to shape the public discourse and public opinion in Hungary before the last parliamentary elections in 2022^12^, resulting in a landslide re-election victory although the opposition led the polls a few months before. An illustrative example of the pro-government influence on the media is that in early 2023, the Breaking News section of the pro-government Origo (Hungary’s third most popular news portal) was subjected to thematic analysis^13^. In their analysis, 727 articles were examined, which were published during the first year of the Russian-Ukraine war. These headlines suggested that Ukraine and the United States were the aggressors, portrayed Zelensky as crazy and drunken, and portrayed Putin as competent and trying to avoid escalation. The headlines echoed the language of Soviet “peace movements” from the Cold War era, using simplistic language and almost exclusively focusing on the war, and repeated widely debunked disinformation claims from the Russian propaganda machinery^13^. Due to this political influence, Hungary has been listed as a “partly free” country since 2019^14^ and moved from the 25th position in 2009 to the 85th position in 2022 on the global list of media freedom^11,15,16^.

In sum, the media situation in Hungary is that of David (independent, mainly liberal media) and Goliath (state-sponsored, pro-governmental, conservative populist media) in terms of resources^11^. In this media context, the bulk of information that lands daily in citizens’ printed journals, smartphones, televisions, and radios derive from pro-governmental sources. In this environment saturated with disinformation, it is an equally critical goal (1) to avoid making people skeptical of real news content and (2) to make them willing to identify misinformation on the dominant channels.

Before we turn to the details of the present research, it might be helpful to distinguish some terms regarding misleading mass communication. The current work defines fake news, disinformation, and misinformation based on Pennycook and Rand^17^. *Fake news* is related to the news content published on the internet that aesthetically resembles legitimate mainstream news content but that is fabricated or highly inaccurate. In the present work, we will refer to fake news as the materials we used in the experimental materials. *Misinformation* and *disinformation* are false or erroneous information but differ primarily in the intent behind their creation and dissemination. More precisely, in both cases, the nature of this news is untrue, inaccurate, or misleading. Misinformation was not necessarily created to deceive, while disinformation was created deliberately to mislead people. Therefore, misinformation is a broader term, including disinformation, characterized by its malicious intent. Some examples of misinformation can be errors in news reports or misunderstandings in shared social media, while examples of disinformation can cover intentionally manipulated hoaxes or propaganda.^17^.

### The present research

Hungary may be the canary in the coal mine. Canaries are more sensitive to toxic gases than humans. If a canary dies, it indicates imminent danger to miners. Young and fragile democracies such as Hungary can be more sensitive to misinformation campaigns than more robust ones. The situation in Hungary is not an isolated phenomenon^9^ but an indication of a concerning trend, and unique within the European Union^4^. Keeping this in mind, in the present article, we provide insights into the gravity of the misinformation problem in Hungary (Study 1), and we introduce a novel intervention approach that builds on the social motivations of Hungarians, equipping them with tools to recognize political fake news and distinguish them from real ones (Study 2).

## Study 1. The gravity of the misinformation problem in Hungary along with political preferences

Social conservative voters seem to be using less of their cognitive capacities while consuming news. A summary of 11 American studies found weaker analytical thinking among political conservatives, compared with liberals, and this was not related to the intellectual capacities of conservatives but rather their motivation to use these cognitive capacities^18–20^. Is this true for Hungarians as well? In Study 1, people rated the credibility of fake and real news which enabled us to compute their score to discern misinformation and to compare people by political leaning.

### Methods

#### Participants

A reputable polling company—that made the most accurate predictions in the past three elections—gathered a sample representative of the country’s online population (*N* = 991, *M*_*age*_ = 50.23, *SD*_*age*_ = 16.07, range = 18–91 years; 51% female; 46.8% high school, 32.5% received a post-secondary degree) who use the Internet at least once a week in April 2021. Among them, 38.6% were supporters of the Orbán government, in line with other representative polls at that time. Participants were requested to take part in an online survey via email and they responded through an online survey platform. They were selected randomly from two internet-enabled panels, including 520,000 members. The panel has been recruited through several channels, both online and offline, such as online advertising on social media and recruitment of respondents to large sample offline surveys. Based on gender, age, type of settlement, and level of education, this sample was representative of those Hungarians who use the internet at least once a week. The sample was created using a multiple-step, proportionally stratified, probabilistic sampling method. The representativity of the sample was ensured by a multidimensional weighting procedure based on the official census data of the Central Statistical Office.

The study received IRB approval at Eötvös Loránd University in accordance with the Declaration of Helsinki and with the informed consent of the participants.

### Materials and procedure

Following the protocol of Pennycook and Rand^21^, participants received 15 factually fake and 15 real news headlines published on a Hungarian fact-checking site (Urbanlegends.hu) or in mainstream news sources (e.g., HVG.hu, Index.hu). One-third of the headlines were *pro-Orbán* (ideologically consistent for conservative and populist government supporters [analogous to pro-Trump news]), e.g., “*We [Hungary] will be among the best 5 countries after the migration crisis has subsided. 2030 will be the great rise of Hungary?”*; one-third were *anti-Orbán* (worldview consistent for supporters of the mainly liberal opposition [analogous to anti-Trump news], e.g., *“Péter Szijjártó [Hungarian Minister of Foreign Affairs and Trade]: “It is treason to protest when migrant hordes are besieging Hungary”. People were outraged at the Foreign Minister*’*s words.*), and one third were politically neutral (e.g., *“The world is celebrating: a diabetes vaccine has been officially announced. It also helps those already suffering from the disease because it reverses the process.”*). Half of the headlines in each category were fake and half were from real news sites. They were presented as screenshots from a Facebook News Feed including a picture, a headline, and a byline. The perceived accuracy of the headlines was measured with the following question: *“To the best of your knowledge, how accurate is the claim in the above headline?” Not at all accurate/not very accurate/somewhat accurate/very accurate*. The order of the news content was randomly presented to participants and all headlines were pre-tested before the data collection. The descriptive statistics can be found online in Supplemental Materials Table S1.

Pro-governmental versus pro-opposition attitudes were measured with the following question: *“If you had to choose between the government and the opposition, which side would you prefer to vote for? The government/The opposition”*.

### Analytic strategy

Using OLS regressions, we compared fake news discernment scores (raw accuracy ratings of real news minus fake news) between pro-governmental conservatives and anti-governmental, mainly liberal, Hungarians.

### Results and discussion

Pro-government voters had lower average discernment scores (average accuracy ratings of real news minus average accuracy ratings of fake news) than their anti-government counterparts, *b* = 0.23 [SE = 0.03], *t*(989) = 8.57, *p* < 0.001, *d* = 0.54, BF_10_ = 1.114 × 10^14^. Specifically, compared to their anti-government peers, pro-government voters evaluated fake news as being more accurate, *b* =  − 0.09 [SE = 0.03], *t*(989) =  − 3.33, *p* < 0.001, *d* = 0.10, BF_10_ = 16.72, and in this distorted media context, they did not believe in real news as they provided lower accuracy ratings on real news content, *b* = 0.14 [SE = 0.03], *t*(989) = 4.70, *p* < 0.001, *d* = 0.54, BF_10_ = 3498.44. It appears that pro-governmental voters have at least as big issues with the correct evaluation of real news as with spotting fake news compared to their anti-governmental peers. The magnitude of difference remains almost completely untouched between pro-governmental and anti-governmental voters if we control for gender, age, level of education, analytic thinking, and digital literacy. The difference remains strongly significant, and the Cohen *d* drops only by 0.04–0.05 (approximately 10% of the effect) after inserting these psychological and sociodemographic variables as covariates.

**Figure 1:**
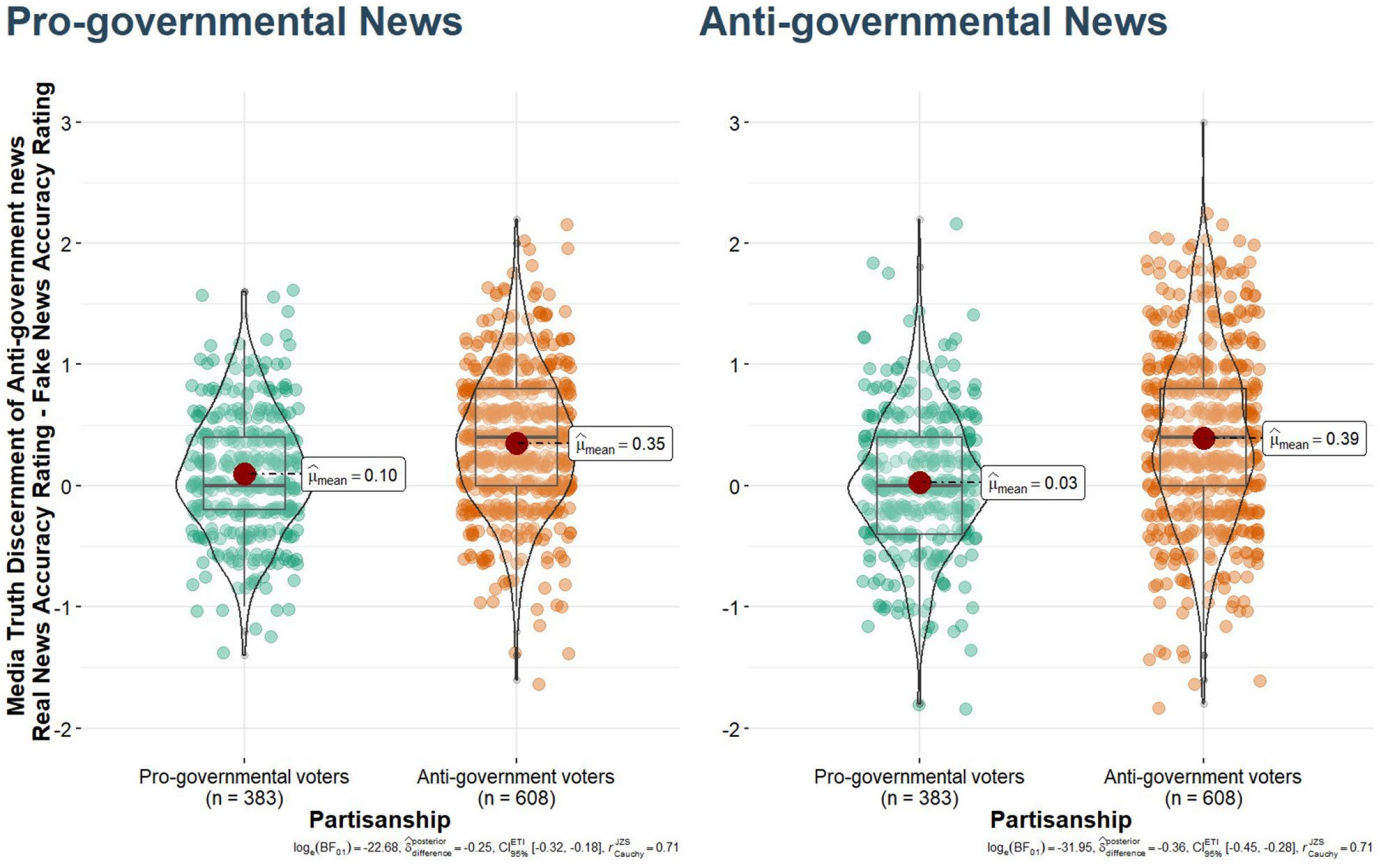
Accuracy rating of real news minus fake news along pro-governmental versus anti-governmental orientation and news type on a representative sample. Based on a representative sample, conservative Hungarian pro-government respondents (green dots) rated fake news almost as accurate as real news on average, however, anti-government voters (orange dots) can distinguish real from fake news better. The left panel displays the means of discernment of pro-governmental news headlines, whereas the right panel displays anti-governmental news headlines in terms of mean real news minus mean fake news evaluations. The scores close to zero mean that the respondents evaluate fake news as correctly as real news, meaning they can hardly distinguish them from each other. Fake and real news were rated on four-point scales; therefore, media truth discernment ranged between − 4 and + 4. On this scale, pro-governmental respondents (green dots) had 0.03–0.1 mean differences between real and fake news headline evaluations, meaning they could hardly distinguish fake from real news headlines. The mean difference was 3.5–13 times larger among anti-government voters.

This difference did not simply reflect a striking split by pro-governmental vs. pro-opposition attitudes, but it also demonstrates that populist conservative voters rated fake news almost as accurate as the real news on average (this is why their value in Fig. 1 is so close to 0)—while anti-government, mainly liberal voters rated real news as more accurate than fake ones (see Fig. 1). This difference was strongly significant and salient in the case of the different news contents such as regarding the pro-government, *b* = 0.25 [SE = 0.04], *t*(989) = 7.25, *p* < 0.001, *d* = 0.46, BF_10_ = 4.165 × 10^9^, and anti-government, *b* = 0.37 [SE = 0.04], *t*(989) = 8.55, *p* < 0.001, *d* = 0.54, BF_10_ = 7.52 × 10^13^, media truth discernment.

In the US, Pennycook and Rand^21^ found Democrats are significantly better at discerning misinformation from real news than Republicans. In Hungary, we found very similar results on a representative sample. Media truth discernment was lower among pro-governmental than anti-government voters. The present study cannot answer why pro-government voters have a harder time differentiating between fake and real news. Still, previous results suggest that analytic thinking moderates the effect of political orientation on media truth discernment, meaning that Hungarian anti-government voters can benefit more from analytic thinking in terms of distinguishing fake from real news compared to pro-government voters^22^. However, another explanation emphasizes that these differences might occur because right-wing voters are exposed to more misinformation^23^. In light of the Hungarian media landscape’s transformation since 2010, this might not shock everyone, but the gravity of the misinformation problem among conservative, pro-governmental voters raises questions: What can social psychologists do in this situation? In particular, how is it possible to design a long-lasting counter-misinformation intervention that resonates with people who are continuously bombarded by the propaganda of an asymmetrically polarized informational autocracy? As the media coverage of pro-governmental channels is overwhelming compared to alternative channels^4,11^, in this media environment, people must develop a selective skepticism in which they are not skeptical of real news, but they can spot misinformation.

## Study 2. A prosocial intervention to motivate Eastern Europeans to spot political misinformation

Various approaches aim to boost people’s resistance to misinformation. Most focus on supporting the individual’s cognitive skills or enhancing their perceptiveness, which leads to immediate or short-term effects that quickly fade. For example, prior ‘nudging’ interventions, that prime people with a simple question: *“To the best of your knowledge, is the claim in the above headline accurate?”* made readers slow down and motivated them to engage their cognitive capacities in the evaluation of upcoming headlines^24^. This accuracy-nudging intervention proved to be broadly effective^25^ and easily scalable on social media sites^26^. Other approaches aim to develop or inoculate digital skills and competencies that try to impart strategies for spotting misinformation. Though they have very promising results in terms of short-term detection of misinformation; however, only one of these studies could demonstrate detectable effects in recognizing manipulative content after one month without boosters, while also meeting important criteria for scalability (Study 5^27^). Furthermore, there is evidence indicating that inoculation increases overall skepticism, causing individuals to place less trust in both real and fake news, rather than enhancing their capacity to identify misinformation^28^.

In a prior intervention, building on prosocial values, we found that young adults could spot misinformation more effectively one month later^7^. In the present study, we will reanalyze this data by focusing on the political aspects in terms of political orientation, politics-related news, and participants’ willingness to use their cognitive capacities. Before detailing the psychological mechanisms leveraged in our intervention, we would also like to set the Hungarian socio-cultural context in which this novel, prosocial intervention proved to be effective.

### Aligning with the existing cultural and historical context of family-based prosocial motivations

Hungary is not exceptional among Eastern European countries concerning its value structure: family- and security-related values have been identified as central to the population since their first measurement dating back to the 1960s^29–31^. Family and the home gained special importance after WW2 and into the 1950s when the ruling Soviet Union-backed socialist party banned more than 90% of clubs, unions, and organizations not under direct political control^32^. In the 1960s and 1970s, people were allowed to make decisions more autonomously in certain parts of their private lives, but any sort of social organization that could potentially challenge the ruling ideology was suppressed or banned. Significant efforts were made to depoliticize society and make people focus on their narrow social groups and compatible family-related or hedonistic values without allowing them to freely organize broader social groups. By the 1980s, the retreat to private life had reached extreme levels. In 1982, 85% of Hungarians said they “would not sacrifice themselves for anything besides their family”—the corresponding figures for Ireland, Denmark, and Spain were 55%, 49%, and 38%, respectively^32^.

Following the fall of the Soviet Union and the transition from socialism to capitalism in 1989, financial security and uncertainty avoidance became more important for Hungarians, but the central role of the family did not diminish^33^. Between 1978 and 1998, the safety of the family was consistently found to be the first or the second most important value people endorsed. In brief, Hungarian society never recovered from the disintegration of social ties in the socialist era, and various political regimes used this decay for their benefit instead of systematically investing in the reconstruction of a social fabric that could support collective values (and altruism that transcends the boundaries of the ingroup). The present work demonstrates how these values can still be leveraged to bring about positive social outcomes that benefit the collective: how the motivation to protect an elderly relative can make the youth more vigilant to spot misinformation.

### Family-based prosocial motives to identify misinformation

Can these family-related prosocial values be capitalized on to encourage people’s discernment of misinformation? Prosocial values can provide a powerful motivational force as people can be more motivated to do extra work for others they care about in contrast to themselves^34,35^. For example, healthcare professionals’ hand hygiene behavior can be more effective if they are reminded of the positive implications for their patients and not for themselves^36^. In the field of education, prior studies showed that *prosocial motives* could help students perform well in monotonous and boring jobs in the US^37–39^. In these interventions, students combined self-oriented and self-transcendent (prosocial) goals to be more persistent. Prosocial goals could pertain to close relations (e.g., their family members, friends) or members of the broader community (e.g., work colleagues, people living in the same town, or everybody on the planet). Considering that Hungarians can be motivated to make an extra cognitive effort for their loved ones, we decided to focus on close relatives and the responsibility to help the digitally vulnerable elderly members of the family.

### Additional psychological mechanisms supporting prosocial motives

#### Digital expert role

Unlike most prior misinformation interventions, that put people in a ‘learner’ role (implicitly assuming relative incompetence), our approach granted participants a digital expert role within their families. One of these mechanisms was attributing a stable and positive digital expert role to the participants which derived from their young generational status compared to their elderly family members’ generational status^40^. The intervention framed youth vigilance to online misinformation as a role-modeling behavior for aiding the digitally less competent older generation. Both adolescents and young adults are sensitive to *social-status-related signs,* so we aimed not only to highlight their expertise but also to demonstrate how digital responsibility can be a source of respect and higher status in their peer groups and beyond^41^.

A *learning mindset perspective* provided a general framework for this intervention (similar to^42^). It means that we framed the digital strategies as competencies that can be developed through (1) making an effort, (2) choosing elaborate learning strategies, and (3) asking for advice from more competent people. An effort is needed as there are barriers that can prevent the spotting of misinformation, such as fatigue or a wandering mind. The core of the intervention material was based on a list of strategies^43^ that help people identify misinformation. Participants were encouraged to learn about these strategies through the testimonials of fellow students.

**Figure 2:**
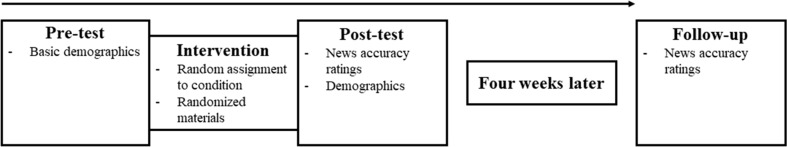
Timeline.

A *self-persuasive exercise* followed the testimonials similar to the prosocial purpose intervention of^39^. In writing a letter to their elderly loved ones, youngsters were supposed to indirectly persuade themselves, which can lead to longer-lasting effects than direct persuasion^44^. Explaining the strategies can also induce hypocrisy by highlighting the distance between the advice and their behavior, which also motivates them to behave in accordance with their advice^45,46^ (see the simplified timeline of the study in Fig. 2). Please find more details regarding the intervention content and the relevant mechanisms in Orosz et al^7^.

In a randomized controlled trial, we found that these psychological mechanisms can help people spot misinformation^7^. However, this earlier intervention work did not focus on the political aspects and implications at all. Therefore, the question arises: Are these benefits of the intervention prominent if we focus only on political news and especially on the distinction between pro-governmental fake from real news?

### Conservative political side and vulnerability to misinformation

Though misinformation poses a potential threat to the whole of society, there are social groups that are especially vulnerable to the harmful effects of misinformation. Research from the US and Western Europe^47^ dominantly shows that conservative voters are more likely to be deceived by misinformation^48,49^ due to their heightened susceptibility to threatening information^50,51^ and less deliberative cognitive styles compared to liberals^19^. Based on Study 1 and international literature from Western countries^48,51^, in the Hungarian context, conservative government supporters are expected to be more vulnerable to misinformation. Our intervention leverages *family bonds* to motivate young adults to build resistance against misinformation, and these family values are more salient in conservative political communication^52^; therefore, we suspect that the intervention can be more effective regarding pro-governmental news content that often highlights the importance of family values.

### Scalable counter-misinformation interventions with long-term effects

Only a handful of studies examined the long-term effects of counter-misinformation interventions and its intersection with scalable ones is even scarcer. These studies applied mainly inoculation^27,53–57^ or other forms of competency-fostering cognitive techniques (e.g., digital media literacy interventions^43^). Inoculation-based and competency-fostering cognitive techniques require people’s cooperation and in-depth engagement^58,59^, therefore they are hardly scalable^17^, and sometimes they take from half an hour to several hours, which makes them difficult to implement among the general public. However, a more recent attempt to translate these approaches to video formats may address the issue of scalability^60^. Unfortunately, without boosters, the immunizing effects of inoculation and competency-fostering interventions either vanish completely in the long term (e.g., effect sizes dropped from *d* = 0.13 to *d* = 0.05 in India^43^) or decreased considerably over a short period (e.g., effect sizes dropped from *d* = 0.95 to *d* = 0.28 within one week and from *d* = 0.20 to *d* = 0.08 in the US; see^43,55^). However, we are not aware of any published studies that found a long-term effect for more than three weeks without boosters. Only two unpublished inoculation intervention studies found a significant effect after 29 days (see the preprint of Maertens et al.,^27^). The first was a written, less scalable inoculation intervention in which the outcome measure was the rating of the perceived scientific consensus among scientists on human-induced climate change (Study 1^27^, based on^55^). The second study was a video-based inoculation task, in which the outcome was discerning manipulative and non-manipulative content (Study 5^27^, based on^60^). In both cases, the effect sizes were *d* = 0.28 (written inoculation) and *d* = 0.23 (video-based inoculation). In sum, despite the strong interest in durable counter-misinformation interventions, very few studies aimed to assess long-term effects and among them, there was only one scalable intervention among these that found effects enduring over four weeks without any sort of boosters (Study 5^27^). These interventions were assessed in a media context where the mainstream media channels do not spread misinformation daily.

### The present research project

Digitally vulnerable people need scalable interventions, which not only change people’s behavior in the short term but ideally make a lasting difference. Our first goal was to design an intervention that can be effective beyond Western and independent cultural contexts where misinformation is peripheral compared to a context where the government controls most media platforms and is the most important source of systematic misinformation^61,62^. In light of the results from Study 1, our second goal was to fill this gap and design a scalable intervention with long-lasting effects that not only trains digital literacy skills but also provides good reasons for spotting pro-governmental misinformation without becoming skeptical about pro-governmental real news.

### Methods

This study was conducted with the ethical approval of Eötvös Loránd University, in accordance with the Declaration of Helsinki, and with the informed consent of the participants.

This initial study was pre-registered (https://osf.io/8tgk6); however, in the preregistration, we had more general hypotheses^7^, without any specific focus on political aspects. In the present work, we turn towards these unexplored aspects.

### Participants

Participants of the intervention were students enrolled in various majors at a Hungarian public university and 801 young adults read the randomized intervention or control materials (*M*_age_ = 22.02; *SD*_age_ = 4.11; 73.46% female; 95.33% Caucasian, 34.40% first-gen). They were recruited from a class and, participation in the study was voluntary and all the students who opened the link agreed to participate. There was some attrition in the follow-up, as data was collected in the middle of COVID-19’s fourth wave in Hungary and some students did not provide an appropriate Student ID which prevented us from matching their follow-up to their intervention data. This led to an attrition of 27.72% of students with intent-to-treat follow-up data from 72.28% of the allocated students (*N* = 577, *M*_age_ = 21.98; *SD*_age_ = 3.85; 76.08% female; 96.55% Caucasian, 33.94% first-generation, for a summary see^21^).

### Procedure

Welcomed and briefed on the study, participants first filled out a measure assessing the frequency of social media use and demographics and then proceeded to their randomly assigned condition (please find further details about the protocol in^7^).

For the *treatment group,* the exercise was framed as a contribution to an online media literacy program developed for the parents’ and grandparents’ generation. Participants read about six scientifically supported strategies (all adapted from^43^), accompanied by peer testimonials, that could help one spot misinformation online. Participants were then asked to compose a letter to a close family member that summarized the strategies and to reflect on the best arguments and advice that would persuade their readers to utilize these strategies in life. The strategies included skepticism for headlines; looking beyond fear-mongering; inspecting the source of news; checking the evidence; triangulation; and considering if the story is a joke.

For the *control group,* the exercise was similar in both structure and content but was not related to the news. It was framed as a contribution to a social media literacy program developed for the parents’ and grandparents’ generation. It was related to practices of the parents or grandparents that the younger generation finds especially embarrassing. The structure of the control material was very similar; however, the topic of fake news did not appear. Participants read about six practices violating tacit norms of social media use. These practices included using Facebook’s feed instead of private messaging; virtual bouquets for birthdays and name days; inappropriate emoji use; ‘funny’ profile pictures; inadequate device handling during video calls; and mass invites for online games. They were then asked to compose a letter to a close family member that summarized the practices and to reflect on the best arguments and advice that would persuade their reader to avoid these practices online.

### Measures

#### Fake news accuracy and media truth discernment

Following the well-established protocol of Pennycook and Rand^21^, we captured perceived political news accuracy by having participants rate real and fake news items on a four-point scale, similar to Study 1 (*“To the best of your knowledge, how accurate is the claim in the above headline?” Not at all accurate/not very accurate/somewhat accurate/very accurate*). In the follow-up one month after intervention, there were six real and six fake news items, half of them pro-governmental and half of them with anti-governmental political content. The items overlapped with the item set we also used in Study 1, and all had been pre-tested in a prior culturally adjusted replication study^22^ of Pennycook and Rand^21^. Fake news accuracy scores were related to the accuracy rating of the fake news items, whereas the media truth discernment scores were calculated by subtracting the mean perceived accuracy of fake items from the mean perceived accuracy of real items. Further self-reported and behavioral measures were implemented immediately after the intervention and also in the follow-up (descriptive statistics of these variables can be found online in Supplemental Materials Table S2).

*Pro-governmental versus pro-opposition attitudes* were measured with the same question as in Study 1 (*“If you had to choose between the government and the opposition, which side would you prefer to vote for? The government/The opposition”*).

The timeline of the study is shown in Fig. 2.

### Analytic strategy

Using OLS regression models, we examined the effect of the condition (treatment vs. control) on political media truth discernment scores (real news accuracy average minus fake news accuracy average, based on^17,21^) controlling for political orientation. We ran this analysis for the long-term (one-month) effects together for all political news and also for pro-governmental news and anti-governmental news content separately. We were interested in the differential effect of the intervention on the pro-governmental fake news content leaving intact the pro-governmental real news evaluations. Finally, we analyzed the effect of the intervention separately among pro-governmental and opposition voters.

Our primary analysis was *intent-to-treat* analysis including all respondents who reached the end of the survey and provided data about their fake news accuracy (see the pre-registration: https://osf.io/8tgk6). We could not analyze the responses of those students who dropped out before the outcome measures, as we did not have relevant outcome variables (the accuracy of fake- and real news). For assessing the long-term results, we could only use the data of those respondents who were randomly allocated to the treatment and the control groups and also finished the accuracy ratings in the follow-up. Besides the main effect of the intervention, we were interested in the effect of the intervention among pro-government respondents.

We studied not only the socio-demographical attributes of the participants but also the linguistic characteristics (e.g., elaborateness, style, formulation) of the letters written by them. We applied three-fold cross-validated and fine-tuned XGBoost^63^ models in Python to classify the respondents’ performance on the discernment task. The outcome variable of the models were the discernment scores and average accuracy ratings of real news minus average accuracy ratings of fake news, both pro-governmental and opposition variants. Those were recoded into binary variables to indicate whether a student performed below average. Those participants who performed above the average were encoded as one, and those with lower performance scored zero (see Table S3 and Table S4 in the Supplemental Materials regarding the proportion of respondents belonging to each category for both pro-governmental and opposition scores). XGBoost classification models were built to classify categories for both pro-governmental and pro-opposition discernment binary outcomes separately.

### Results

#### Preliminary analyses and attrition

We examined both samples’ overall attrition (independent of condition) and differential attrition (condition-dependent). Considering the *overall attrition* out of the 16 sociodemographic (age, gender, level of education, first-generation status, parental education separately, place of residence, political orientation), social media use (Facebook-, Instagram-, Snapchat-, and TikTok use), and relevant individual differences variables (need for cognition, cognitive reflection task, pre- and post-intervention bullshit receptivity), we examined in the pre-and post-intervention analyses, we found overall attrition differences (participants who stayed in the follow-up differed from those who started the intervention) regarding gender (*z* = −2.745; *p* = 0.006) and minority status (*z* = −2.925; *p* = 0.003). It means that independently from the condition, fewer male and minority participants were part of the follow-up than in the first session. Although we made a significant effort to design a control condition very similar to the intervention one, in terms of differential attrition, we found one significant difference along gender (z = -2.317; *p* = 0.021). Less female participants dropped out from the follow-up of the control condition than from the intervention condition.

### Primary analyses

#### Fake news accuracy and discernment in general

Overall, the results showed that the intervention produced significant media truth discernment score (mean real news accuracy scores minus mean fake news accuracy scores) changes, *b* =  − 0.13 [SE = 0.05], *t*(545) =  − 2.47, *p* = 0.014, *d* = 0.21, BF_10_ = 0.238 relative to the control condition. As the intervention did not influence the real political news accuracy scores at all *b* =  − 0.00 [SE = 0.04], *t*(545) = -0.02, *p* = 0.981, *d* = 0.00, BF_01_ = 9.87, therefore it did not lead to a general skepticism towards political news; however, it massively influenced the political fake news accuracy ratings, *b* = 0.13 [SE = 0.04], *t*(545) = 3.22, *p* = 0.001, *d* = 0.27, BF_10_ = 8.81. Based on the Bayesian analysis, it is 9.87 more likely that the real news evaluations are not different between the treatment and the control and 8.81 times more likely that the fake news correctness ratings are lower in the intervention group than in the control group. Therefore, the intervention, after four weeks led to a selective change in the real and fake news evaluation.

**Figure 3:**
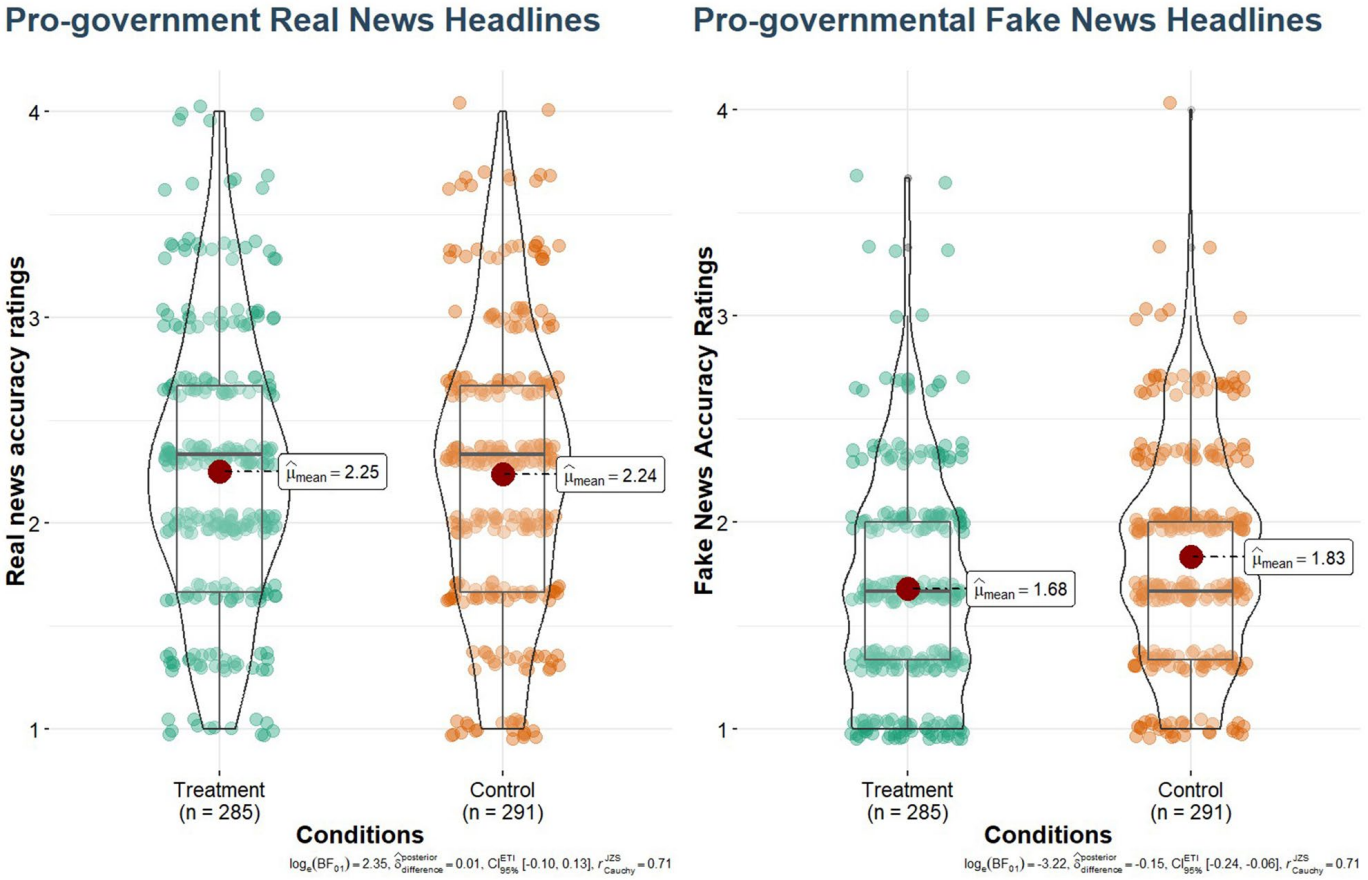
Real and Fake News Accuracy Ratings Regarding Pro-Governmental Political News One Month After the Intervention (raw means). Participants of the intervention benefitted more than their peers in the control group in terms of distinguishing pro-governmental fake from real news. We found a significant difference between the two conditions in the pro-governmental fake news correctness ratings. However, there was no difference in the pro-governmental real news accuracy ratings. On the left panel, the y-axis represents real news accuracy ratings, in which higher values are desired as they indicate the correct evaluation of real news. On the right panel, the y-axis represents the correctness ratings of fake news. Higher scores represent undesired outcomes, indicating that the participants found fake news headlines accurate. The red dot indicates the median, the black line in the middle indicates the mean, the box indicates the interquartile range (between the first and the third quartiles), the curvy shape represents the distributions, and the single dots represent the data.

However, the discernment effect of the intervention on the pro-governmental news, *b* =  − 0.19 [SE = 0.07], *t*(545) =  − 2.76, *p* = 0.006, *d* = 0.23, BF_10_ = 1.485, was two times larger than the non-significant pro-opposition media truth discernment, *b* = -0.08 [SE = 0.06], *t*(545) =  − 1.19, *p* = 0.236, *d* = 0.10, BF_10_ = 0.120. More precisely, the effect of the intervention on the fake news correctness was very solid, *b* =  − 0.36 [SE = 0.05], *t*(545) =  − 1.19, *p* < 0.001, *d* = 0.31, BF_10_ = 25.02, (see Fig. 3 right panel), while the intervention did not change the pro-governmental real news at all, *b* =  − 0.04 [SE = 0.06], *t*(545) =  − 0.42, *p* = 0.676, *d* = 0.04, BF_01_ = 10.44 (see Fig. 3 left panel). These results indicate that it is 25 times more likely that the intervention made people spot pro-governmental news (than the control) and it is 10 times more likely that the participants of the intervention did not become more skeptical about pro-governmental real news (compared to the control).

Finally, when we examined the impact of the intervention on the fake news discernment scores among pro-government voters, *b* =  − 0.17 [SE = 0.10], *t*(154) =  − 1.74, *p* = 0.083, *d* = 0.26, BF_10_ = 0.69, and opposition voters, *b* =  − 0.12 [SE = 0.07], *t*(390) =  − 1.84, *p* = 0.067, *d* = 0.18, BF_10_ = 0.57, we found marginal effects as the present study was underpowered to do such analyses. However, based on the effect sizes one might suppose that conservative, pro-government voters can especially benefit from the intervention content.

### Causal forest analysis to identify conditional treatment effects

Our study’s findings, leveraging a supplementary causal forest algorithm and SHAP value analysis, illustrate a positive and significant impact of the intervention on pro-governmental fake news discernment. Notably, the Conditional Average Treatment Effects (CATE) analysis, supported by the above-detailed OLS regression, underscores the intervention’s effectiveness in enhancing the ability of participants to discern pro-government fake news from real ones. Based on the CATE analysis, this impact is particularly pronounced among older participants, those living in rural areas, males, and pro-government supporters, indicating a targeted intervention’s potential to improve misinformation discernment in specific demographic groups.

Despite some uncertainty indicated by the confidence interval’s lower limit, the overall positive CATE value and supportive OLS regression results confirm the intervention’s sustained effectiveness. This comprehensive analysis, detailed in the Supplementary Material (see Figure S2 and Figure S3), reinforces the main manuscript’s findings on the long-term benefits of our intervention in fostering discernment of pro-government fake news within the Hungarian context.

### The role of potential linguistic cues in the efficacy of the intervention

Besides these analyses, based on the XGBoost classification, we summarized the importance of the potential linguistic predictors assessed (in the light of socio-economic variables). We found that the letter’s elaborateness, the use of conditional mode, and the use of imperative sentences, along with a loving and dutiful writing style, were among the ten most important features for classifying students’ performance on the discernment task. These results suggest that besides socioeconomic features, various letter-writing features were related to the efficacy of the intervention (for further details, see the Supplemental Materials, Figure S4, and Figure S5), and in future intervention studies, emphasizing conditional mode (instead of imperative sentences) and loving kind style can be beneficial to vulnerable target groups.

## Discussion

While these lines are written, we are in the middle of the fifth coronavirus wave and an exploding European energy crisis. Hungary’s neighbor, Ukraine, is under siege by the Russian military, a country with a massive international disinformation arsenal that greatly impacts Eastern and Central European countries^64^. In this context, information over misinformation is crucially important, especially for Eastern and Central Europeans. The goal of the present intervention was to leverage family-oriented prosocial motivations that constitute one of the most important value pillars of Hungarian culture^32^. According to the results, young people—by explaining fake news discerning strategies (e.g., skepticism towards headlines or looking beyond fear-mongering) to their elderly family members—developed a long-lasting digital skill to distinguish fake from real news. These effects held a month later and were particularly high regarding pro-governmental news content without harming the accuracy ratings of the real news accuracy evaluations.

Unlike prior fake news interventions using nudges^17,24,65–69^, inoculation techniques^27,53–57,70–78^, or digital competence building^43,53,74–76,78–82^, the present program addressed *why fake news detection is important*. It could catalyze recursive psychological processes that keep young adults vigilant to discern fake news and the effect of the intervention can remain over a longer period. Prior fake news interventions very rarely found long-term effects (e.g.,^7,27^). In contrast to these prior studies, our study found an effective tool for reducing the susceptibility to (pro-governmental) conservative fake news without any boosters. We believe that the main reason for this is that prior interventions focused on the individual and their cognitive motivations but did not use the motivations of relevant social bonds.

Despite using social motivations, it does not mean that the cognitive changes are unexpected or could not happen. On the contrary, these processes could provide ground for habit formation and habit change in which there is no need to suppress unwanted behaviors^83^. Future studies might explore exactly how these interventions can have an impact on the long-term cognitive processing of news content as well as their underlying neural structures.

## Theoretical contributions

How can psychologically wise interventions^84^ contribute to misinformation research? Instead of competing among nudges and inoculation techniques or competency-building interventions, how is it possible to combine the best of these approaches to advance the fight against misinformation?

All these approaches put the individual at the heart of the intervention, but it does not mean that they cannot incorporate a framing of interdependence. Nudging interventions^17,24,65–69^ might be combined with these techniques to remind people of their expert role and responsibility toward elderly relatives. Accuracy nudging^17,24,65–69^ can be effectively implemented not only to make people temporarily elaborative right after the intervention, but they can remind people and reinforce the narrative and the reasons why it is important to be vigilant to spot misinformation. In inoculation and competency-building interventions^27,43,53–57,70–82^ emphasizing the expertise of the participants can reduce reactance and can provide an additional layer of motivation and persistence to incorporate the intervention materials (instead of putting them in a more or less inferior position in which it is assumed that they are digitally incompetent^58,59^.

On the other hand, inoculation interventions could add prosocial motives by encouraging to explain to loved ones the reasons why it is important to play the fake news game. However, in these works, it might be also essential to consider the cultural and social-class-related values. The Hungarian context provided excellent ground for the harnessing of interdependent, family-based prosocial values^33^; yet, in other countries (e.g., in the USA) prosocial motivations towards broader communities or more specific identity-relevant social groups (such as ethnic, class, and neighborhood-related groups) might be more effective to motivate people to spot misinformation^38,39^.

No interventions can resonate with every narrative^85^. In Hungary—based on dominant societal values—the protection of families is a fundamental part of the political narrative, especially recently. The country has no ministry of education or health care, but there is a minister for families—the former one was inaugurated as the president of the republic earlier in 2022. Based on the massive governmental communication, Hungarian families receive various sorts of psychological threats and readymade protection from the state. For example, the government is tamping constantly the “threat of migrants” (asylum seekers in 2015), the threat of George Soros, the threat of Brussels, the threat of high utility bills (etc.), and they provide paternalist protection to these threats. Most importantly, the government’s communication resonates very well with the dominant value of society: the protection of families. For these reasons, we found that there is a salient effect regarding fake news aligned with the messages of the pro-Orbán conservative political party.

## Applied contribution

Researchers and policymakers hope for interventions with optimal scalability, long-term effects, and solid efficacy on vulnerable groups^17^. Yet, immediate implementation to wide audiences is usually difficult as the framing of these interventions is critical. Our approach was effective among young adults in the context that made it plausible for them to give advice that helps the preparation of an intervention for elderly people. We may achieve similar effects by scaling this program through online adverts and getting many young adults to write kind letters to their elderly relatives. A media campaign for promoting the intervention and supported by private companies (with the support of a responsible tech company) and public agencies (with the support of national agencies responsible for life-long and digital learning) can be an approach that can reach the masses on both sides of the political spectrum, especially in these times when there is strong social media consumption.

From our perspective, the value structure of the other Eastern European countries is not very different from the Hungarians^86^. After WWII, the Soviet occupation made significant efforts to cut off extra-familiar bonds not only in Hungary (for a study on Poland see^87^) as the spontaneously emerging social connections and groups could pose a potential danger to the Soviets’ political power. Similarly to Hungary, the consequence of these atomizing policies in terms of anomic wounds has not been healed in these countries^32^. Hence, the narrow scope of prosocial intentions (focusing on friends and family) holds in other countries of the region, suggesting that misinformation interventions could successfully capitalize on these familial bonds. Future studies might explore the application of this intervention content in other cultural contexts.

The XGBoost analyses suggest that this intervention can be considerably improve if in future studies we can make participants focusing on the elaborateness, formulation, and style of the letter. For example, if we can design the saying-is-believing task in a way that it conveys elaborated messages lovingly and kindly by using conditional mode instead of imperative one, it might be possible to increase the efficacy in discerning fake from real news.

### Limitations

Despite being a randomized controlled trial with carefully pretested behavioral measures, the present work is not without limitations. First, despite we used a very precisely matching active control group, we identified mild differential and overall attrition differences. As it was among the first steps of the intervention development, our sample was not nationally representative and comprised university students. We cannot, therefore, extrapolate how effective this intervention among young adults is in the general population. Even though we asked five Hungarian polling companies, unfortunately, none of them could guarantee even a 40% (or lower) attrition rate independently from the rewarding structure, or even declined our request. Therefore, we had no other choice than to apply it to a non-representative sample where we could reliably receive follow-up data. From this perspective, the university sample appeared to be reasonable, in which we could guarantee motivated participants with an appropriate attrition rate. It was only tested in the Hungarian context; we do not know yet whether the intervention is similarly effective in other Eastern and Central European countries. Finally, similar to almost all interventions, as a dependent variable, we did not measure the actual social media behavior over time but used Pennycook and Rand’s^21^ assessment method.

## Conclusion

Misinformation interventions can save lives in pandemics and wartimes—both of which have recently been witnessed by Central and Eastern Europe, a region that is re-emerging as a political and informational buffer zone between Russia and the Euro-Atlantic alliance. The core psychological motive underlying our intervention, prosocial family values, was inspired by the theoretical and methodological approach of wise interventions^39,84^, but also influenced by the communication strategies of the populist Hungarian government that often builds on similar values to rally its electorate (e.g., when they frame asylum seekers as threats to Hungarian families). The present intervention, framed around the protection of vulnerable family members, demonstrates that these values can be leveraged to boost critical thinking and misinformation discernment and could be highly effective in cultures where interdependence and family values are important. This intervention might be only a first step towards a new generation of misinformation interventions that combine prior individual-focused strategies with social ones.

### Supplementary Information

Supplementary Information.

## Data Availability

Study 1: The dataset that supports the findings is openly available on Open Science Framework (DOI10.17605/OSF.IO/VCF36) and can be found here: https://osf.io/vcf36/. Study 2: The dataset that supports the findings will be openly available on Open Science Framework (DOI [submitted to the present manuscript]) and can be found here.
