# Supplementary material for: Softly empowering a prosocial expert in the family: lasting effects of a counter-misinformation intervention in an informational autocracy
Source: Sci Rep. 2024 May 23;14:11763. doi: 10.1038/s41598-024-61232-x (PMC11116454; doi:10.1038/s41598-024-61232-x)

**Supplemental Materials**

**for**

**Softly Empowering a Prosocial Expert in the Family: Lasting Effects of a Counter-Misinformation Intervention in an Informational Autocracy**

Content

**Table S1: Descriptive Statistics of Fake and Real News Headlines (Study 1)2**

**Figure S1: Media Truth Discernment as a Function of Political Ideology and Political Leaning of the Headline (Study 1)3**

**Table S2: Descriptive Statistics of Main Variables (Study 2)4**

**Intervention Material – Treatment Group (Study 2)5**

**Measures Used as Covariates (Study 2)10**

**Causal Forest Analysis to Identify Conditional Treatment Effects** **(Study 2)11**

**The Role of Potential Linguistic Cues in the Efficacy of the Intervention (Study 2)13**

**Table S1: Descriptive Statistics of Fake and Real News Headlines (Study 1)**

|  | *N = 991* | |  |
| --- | --- | --- | --- |
|  | *M* | *SD* |  |
| Accuracy of pro-government fake news | 1.97 | .66 |  |
| Accuracy of pro-government real news | 2.22 | .59 |  |
| Accuracy of anti-government fake news | 2.28 | .63 |  |
| Accuracy of anti-government real news | | 2.54 | .70 |
| Accuracy of politically neutral fake news | 1.90 | .53 |  |
| Accuracy of politically neutral real news | 2.37 | .56 |  |

*Note:* The acceptance of fake and real news was measured with a scale from 1 (not at all accurate)
to 4 (very accurate), higher means indicate higher perceived accuracy.

**Figure S1. Media Truth Discernment as a Function of Political Ideology and Political Leaning of the Headline (Study 1).** Supporters of the opposition were considerably better at discerning fake from real news (irrespective of the type of news) than supporters of the government. Error bars represent 95% CIs.

*Notes.* A high media truth discernment score indicates an increased capacity to distinguish real from fake news. “Government supporters” is rather similar to “Republican voters” in the US context, while “Voters of the opposition” is an analogy with “Democrat voters”.

**Table S2: Descriptive Statistics of Main Variables – Study 2**

|  | Intervention | | Control | |
| --- | --- | --- | --- | --- |
|  | *Raw M* | *SD* | *Raw M* | *SD* |
| Accuracy of political fake news – immediately | 1.83 | 0.53 | 1.99 | 0.52 |
| Accuracy of political real news – immediately | 2.58 | 0.55 | 2.56 | 0.52 |
| Accuracy of political fake news – follow-up | 2.00 | 0.50 | 2.12 | 0.48 |
| Accuracy of political real news – follow-up | 2.50 | 0.52 | 2.51 | 0.52 |
| Pro-governmental accuracy of political fake news – follow-up | 1.68 | 0.55 | 1.83 | 0.53 |
| Pro-governmental accuracy of political real news – follow-up | 2.25 | 0.67 | 2.24 | 0.69 |
| Bullshit receptivity – pre-intervention | 2.42 | 0.91 | 2.37 | 0.89 |
| Bullshit receptivity – post-intervention | 1.98 | 0.81 | 2.08 | 0.82 |
| Bullshit receptivity – follow-up | 2.19 | 0.87 | 2.28 | 0.83 |
| Cognitive reflection – immediately post-intervention | 3.06 | 1.44 | 3.12 | 1.33 |
| Need for cognition – immediately post-intervention | 4.93 | 1.11 | 4.86 | 0.99 |
| Digital literacy – follow-up | 2.28 | 0.66 | 2.29 | 0.62 |
| Conspiracy mentality – follow-up | 66.10 | 14.70 | 68.10 | 14.84 |

*Note:* The acceptance of fake and real news was measured with a scale from 1 (not at all accurate)
to 4 (very accurate), higher means indicate higher perceived accuracy. Bullshit receptivity scales were measured using 5-point Likert scales (1 – not at all profound; 5 – very profound): the higher the mean, the greater bullshit receptivity is. Cognitive reflection items were coded as correct (1) and wrong (0), and a higher mean represents higher analytical thinking. We used 7-point Likert scales to measure Need for cognition (1 – I don’t agree at all; 7 – I completely agree): the higher the mean, the greater the need for cognition. Answers to the Digital literacy items ranged between 1 (never) and 5 (very often), lower means indicate higher digital literacy. Conspiracy mentality was measured on a scale from 1 (low conspiracy mentality) to 11 (high conspiracy mentality): the higher the mean, the higher the conspiracy mentality is.

**Intervention Material – Treatment Group**

All materials appeared in Hungarian for the respondents.

**Slide 1**

**What should we teach our parents and grandparents about reading the news?**

We are developing a new program for your generation of parents and grandparents.

We would like to ask you to help with this development! The program presents scientifically based strategies on what to pay attention to filter out fake news.

Example strategies:

- Be skeptical of the titles

- A closer look at the source of the news

- Read other reports

If we can learn from your thoughts on how to present those strategies, this program can benefit your parents and grandparents.

**Slide 2**

**Why did we create the program?**

We are researchers at *Anonymous University*, and we created this program because many older people want clearer guidance on how to navigate the world of online news.

However, this requires even more real examples from you, from young people who are very familiar with the online world and know exactly what messages are coming to your parents and grandparents about online things.

Even though we know effective strategies to filter out fake news based on well-founded facts, we’re not the experts on how we can explain these messages to them as effectively as possible, it’s you.

**Slide 3**

**What strategies are worth dealing with the news?**

This program presents six scientifically sound strategies to help spot fake news:

• Be skeptical of the titles

• Question the outrageous information

• Investigate the source of the news

• Check the evidence

• Find more articles

• Think about whether the story is a joke

Each strategy can be fully mastered and developed by anyone.

The above strategies have been formulated by independent researchers at Princeton University.

**Slide 4**

**Strategies in the hands of young people**

These strategies have been used regularly by more and more young people in recent years, but there would be a great need for the older generation to be able to apply them routinely as well.

In the following, you can learn some more details about these and why young people consider it important to use their brains when reading the news.

We gave young people these strategies and asked them what they thought of them. In the following, we would like to share some quotes they have written with you.

**Slide 5**

Original strategy:

**Be skeptical of the titles:** If the shocking statements in the title sound incredible, they probably are.

Anna's opinion:

“I find it very embarrassing for someone to share content whose title feels fake. I’m a little more lenient with my mother’s age group, but when it comes to my generation, and especially when someone is close to me, I’m ashamed of myself instead. Last time, I wrote to one of my cousins not to read and use his cell on autopilot, but to use his brain and delete the silly news he shared until he became ridiculed for it. He took it off, and I was proud to post fewer and fewer doubtful things afterward. You don't need any special skills to do this. Anyone who can read can pay attention to it; over time, it can more and more easily filter out things whose titles are already stinky. My parents tell me to be sane when I go to a party... That’s all I can advise them when they press their phone!!”

Anna, a 17-year-old high school student

**Slide 6**

Original strategy:

**Question the outrageous information**: If you are reading something intimidating, ask yourself the question: Was this shared to make me feel that way? If the answer is yes, think carefully about whether you share it.

Tibi's opinion:

“I used to be scared about all sorts of things I read online. For this reason, I have figured out that if a news story comes across with some threatening message, I will stop for a moment and ask myself: Tibi, can this hurt you now or is it unfounded nonsense that was written just to make you scared? Asking this question and thinking about the answer will protect me from being afraid of a lot of unnecessary things. If I even feel a little that a terrifying text was written by someone to make people like me afraid and not because there is an actual threat, it makes me very angry. If they want to manipulate me through fears and threats, it makes me super mad at them because of my history studies.”

Tibi, a 21-year-old student (with a major in history and Hungarian)

**Slide 7**

Original strategy:

**Examine the news source:** Make sure the story was published by a source whose confidence can be trusted. If the story comes from an unknown organization, try to find out more about who they are.

Fanni's opinion:

“If I had been asked where I read the news a few years ago, I, like most of my friends, would have said, quite naturally, on Facebook of course. That would not be cool at all today. Of course, there are normal news stories on my Facebook as well, but this nauseating stream of information is also full of junk and fake news. I used to read the news from here without looking at where they came from. Today, I see this as drinking from the sewer. In recent years, like most of my acquaintances, I have searched for news sources that are accepted on a Hungarian and international level and can be trusted for their accuracy. Obviously, we always need our brains, but these pages give me a sure starting point and they already make it easier for me to see where and how the articles of the little shady outlets are distorted. "

Fanni, a 25-year-old hairdresser

**Slide 8**

Original strategy:

**Check the evidence.** Check the author's sources to make sure they are accurate. Lack of evidence or reliance on unnamed experts may indicate false news.

Bence's opinion:

“I’ve always loved understanding the broader questions in life. However, when I was a teenager, I was even less competent in what to look for when I read about broader theories, and I was deceived by a lot of fake news. Some articles describe world-shattering theories using big and smart-looking words. These stories are really interesting at times, they may even seem straightforward and logical, but even when they reach the thrill of Marvel movies, they often have no basis. Step by step I learned that I should stop after a few minutes reading and ask myself: Bence, look at it, what does he say? But why does he say that? What is he basing this on? Who are these experts he mentions? Where did he get your pictures from? Can he be another self-appointed narrator? And if the answers aren’t right, I’ll put it in the box of forgetful fiction.”

Bence, a 23-year-old university student

**Slide 9**

Original strategy:

**Find more articles.** If another news source does not report the same story, this may indicate that the news is false. If the story is reported by more reliable sources, it is more likely to be true.

Ádám's opinion:

“I used to be enthusiastic about sharing the shocking news so that all my acquaintances would know from me for the first time what was going on in the world. Then one day one of my older water polo training buddies, whom I looked up to very much, told me he saw my post and that I should delete it immediately. He said that if you share new and interesting news, they will think you are informed. If, on the other hand, you share fake news without reason, they will look like a dumb kid, even though I don’t think you are. I went back to the pool and was constantly thinking about when I could delete my post. I’ve been sharing a lot of things since then, but before that, I always check several sites to see if it’s true.”

Ádám, a 22-year-old student of economics

**Slide 10**

Original strategy:

**Think of the story as a joke.** Sometimes fake news is hard to distinguish from humor or satire. Make sure the source is not known for parody and that the details and tone of the story suggest that it was just for fun.

Tamás's opinion:

“A few years ago, I was deceived by a piece of news from The Onion and commented on it as if it were true. A few minutes later, one of my friends wrote to me kindly and asked if everything was okay with me... It might have been funny to my acquaintances from outside of the situation, but it was less funny from inside. Everyone reads the news tiredly and superficially, sometimes everyone believes in nonsense, so accidents can happen. It’s like driving a car, no matter how tired you are, you always must be sensible. You can be very smart but if you don’t use your brain while reading the news, it’s not worth much. On the other hand, if you pay attention, you don’t have to be a genius to pinpoint fake news. In any case, this case reminds me to check the weird news, whether it’s from some fake or a joke site.”

Tamás, 21, a computer science and engineering student

**Slide 11**

**Help explain how to read the news to older generations!**

We collect short letters from digitally educated young people to provide the best examples to the older generations.

As you have seen, these strategies are not complicated things. It is still important to draw their attention to them, which can be developed very effectively with some attention and effort.

In a later program, we will want to share some letters (anonymously) with members of your (grand)parents’ generation. **Therefore, we would like to ask you to write a letter along the lines below.**

Your letter:

• Start by naming your family member who would need this information for example: “Dear Mom / Dad / Grandma / Grandpa!”!

• summarize in a few sentences in your own words the strategies you have just read! (You can find their description below the text box.)

• Then think a little about that

o What arguments can you use to tell them the most effective of these strategies that you want in the future?

o What can you advise them to follow these strategies even when you are not there for them?

• Finally, formulate your letter in a way that brings out the most pervasive arguments and brings thoughts and feelings closest to them about how to spot fake news.

There are no right or wrong answers and please don’t worry about spelling. Just focus on conveying your ideas, thoughts, and feelings!

You can write your letter here: (text box)

**Measures Used as Covariates (Study 2)**

Bullshit receptivity was assessed with ten items^1^ such as *“Interdependence is rooted in ephemeral actions”*. Respondents filled out half of the scale pre-intervention, the other half post-intervention, and the full scale again in the follow-up. The response scale ranged from 1 (not at all profound) to 5 (very profound). All the pre- and post-intervention and follow-up measures’ internal consistency was excellent (⍶pre = 0.84; ⍶post = 0.80; ⍶follow-up = 0.90).

Conspiracy mentality was measured in the follow-up with the CMQ^2^ with five items such as *“I think that many very important things happen in the world, which the public is never informed about”*. Respondents rated their agreement with the statements using percentages ranging from 0% (coded as 1) to 100% (coded as 11) with steps of 10%. The internal consistency of the measure was borderline (⍶follow-up = 0.69).

Digital literacy was measured in the follow-up with five items^3^ such as *“I rely on family members to introduce me to new technology”*. Respondents indicated how often these statements applied to them using a scale from 1 (never) to 5 (very often). The reliability of the scale was acceptable (⍶follow-up = 0.70).

Need for cognition was assessed post-intervention using five items from the NFC scale^4^ such as *“It's enough for me that something gets the job done, I don't care how or why it works”* (reversed item). Respondents rated their agreement with the statements with a scale ranging from 1 (I do not agree at all) to 7 (I totally agree). The reliability of the scale was acceptable (⍶pre = 0.75).

Cognitive reflection was measured post-intervention using the CRT^5,6,7^ with five items such as: *“A bat and a ball cost $1.10. The bat costs $1.00 more than the ball. How much does the ball cost?”* Cognitive reflection items were coded as correct (1) and wrong (0).

1. Pennycook, G., Cheyne, J. A., Barr, N., Koehler, D. J., & Fugelsang, J. A. On the reception and detection of pseudo-profound bullshit. *Judgment and Decision Making* **10***,* 549–563 (2015).
2. Bruder, M., Haffke, P., Neave, N., Nouripanah, N., & Imhoff, R. Measuring individual differences in generic beliefs in conspiracy theories across cultures: Conspiracy Mentality Questionnaire. *Frontiers in Psychology* **4**, 225 (2013).
3. Guess, A. M., & Munger, K. Digital literacy and online political behavior. *Political Science Research and Methods,* 1-19 (2020).
4. Cacioppo, J. T., & Petty, R. E. The need for cognition. *Journal of Personality and Social Psychology* **42**, 116–131 (1982).
5. Frederick, S. Cognitive reflection and decision making. *Journal of Economic Perspectives* **19**, 25-42 (2005).
6. Shenhav, A., Rand, D. G., & Greene, J. D. Divine intuition: Cognitive style inﬂuences belief in God. *Journal of Experimental Psychology. General* **141**, 423–428 (2012).
7. Thomson, K. S., & Oppenheimer, D. M. Investigating an alternate form of the cognitive reflection test. *Judgment and Decision Making* **11**, 99–113 (2016).

**Causal Forest Analysis to Identify Conditional Treatment Effects** **(Study 2)**

We utilized the causal forest algorithm, implemented through the EconML library in Python, to reassess the average treatment effects employing a double robust correction method. Given the specific relevance of pro-government fake news discernment within the Hungarian socio-political landscape (and in the light of the regression results reported in the main manuscript), this variable was designated as the primary outcome of our analysis following a process of standardization. The independent variable of interest, namely the treatment condition, was dichotomized into control and intervention groups. The objective of this analysis is to assess the durability of the intervention's impact on the ability to discern pro-government fake news.

**Figure S2. Causal Forest SHAP values summary (beeswarm) plot**


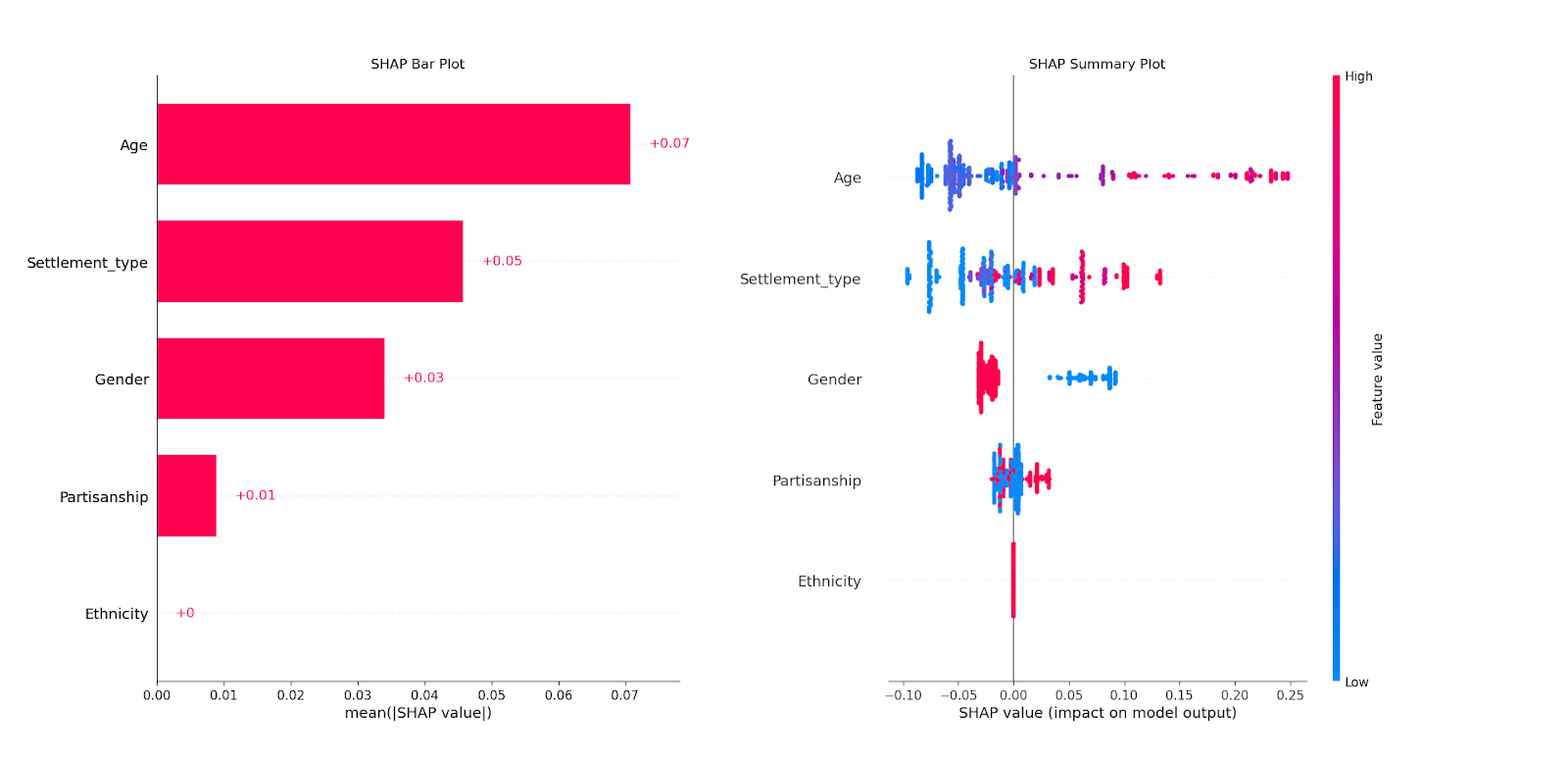


Figure S2 illustrates the SHAP (SHapley Additive exPlanations) values derived from our predictive model, focusing on several demographic and socio-political variables: age, type of settlement (residing in the capital versus the countryside - see below), gender (male versus female), political affiliation (pro-government versus anti-government voters), and ethnicity (members of an ethnic minority versus the ethnic majority). The analysis reveals that age is the predominant factor influencing the discernment of pro-government fake news, with a positive effect size of +0.08. Furthermore, the longitudinal impact of our intervention, as compared to the control condition, on the ability to identify pro-government fake news is notably more pronounced among (a) older participants, (b) those residing in rural areas, (c) males, and (d) individuals who support the government. Detailed SHAP values for each of these demographic and socio-political characteristics are provided below, elucidating their respective contributions to the model's predictions.

**Figure S3. CATE (conditional average treatment effects) and its confidence intervals**


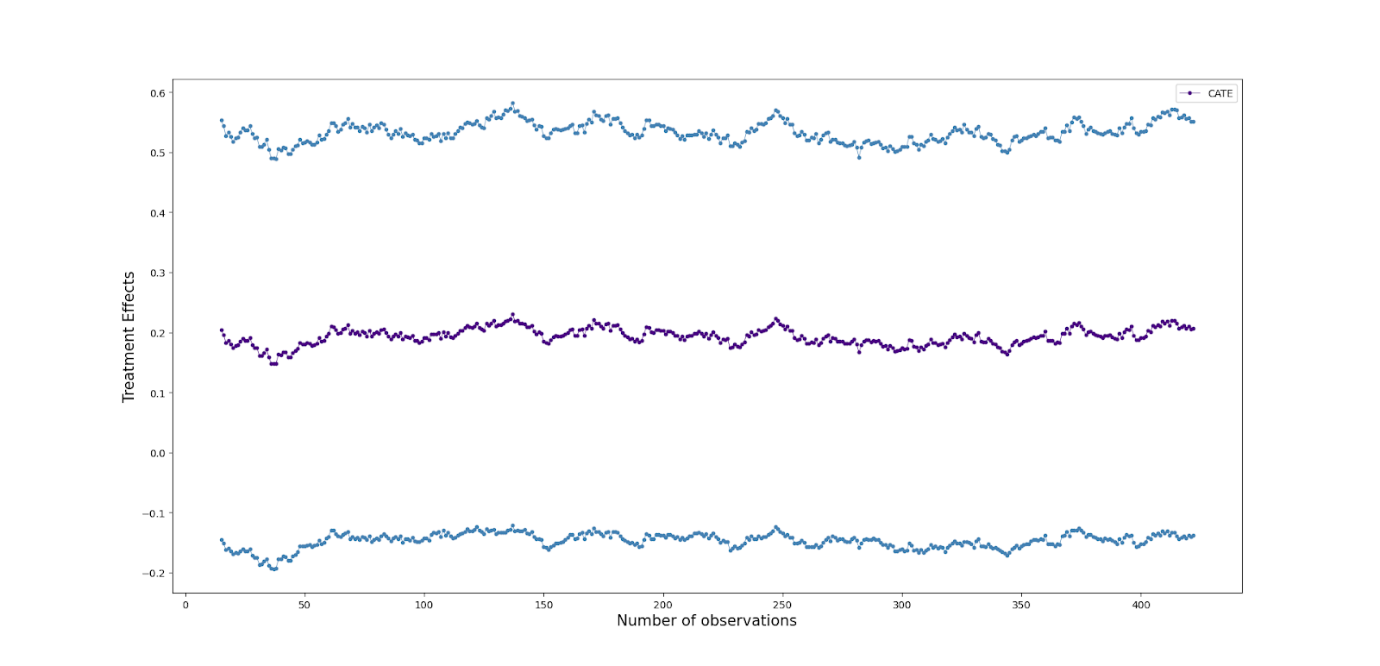


In Figure S3, we present the estimated Conditional Average Treatment Effects (CATE) of our intervention, depicted in deep blue, along with its 95% confidence interval limits, shown in light blue. Despite the lower limit of the 95% confidence interval falling below zero, the positive CATE value derived from the causal forest model suggests that our intervention has a significant, enduring effect on the ability of participants to discern pro-government fake news. This finding is corroborated by the results obtained from Ordinary Least Squares (OLS) regression analysis. While the confidence interval introduces a degree of uncertainty, it does not undermine the overall efficacy of the intervention. Importantly, this analysis reinforces the primary regression findings regarding the sustained effectiveness of our intervention in enhancing pro-government fake news discernment.

Label: Gender

Man: 0

Woman: 1

Label: Ethnicity

Minority: 0

Not minority: 1

Label: cond

control: 0

intervention: 1

Label: Settlement_type

Capital city (Budapest): 0

County seat, city with county rights: 1

Municipality: 2

Other city: 3

Label: Partisanship

Anti-government: 0

Pro-government: 1

**The Role of Potential Linguistic Cues in the Efficacy of the Intervention (Study 2)**

**Methods**

We studied not only the socio-demographical attributes of the participants but also linguistic characteristics (e.g. elaborateness, style, formulation) of the letters written by them and applied 3-fold cross-validated and fine-tuned XGBoost (Brownlee 2016) models in Python to classify the performance of the respondents on the discernment task. The outcome variable of the models were the discernment scores, and average accuracy ratings of real news minus average accuracy ratings of fake news, both pro-governmental and opposition variants. Those were recoded into binary variables to indicate whether a student performed lower or above average; those participants, who performed above the average were encoded as one and those with lower performance on the score with zero (see Appendix Table 1. and Table 2. Showing the proportion of respondents belonging to each category for both pro-governmental and opposition scores). XGBoost classification models were built to classify categories for both variables separately (see Results, for both disc_pro_gov_binary and disc_pro_opp_binary outcomes).

**Results**

Well-aligned with the previously describes results, we found that importance of political attitude (whether the respondent favored pro-governmental or non-governmental parties) was high, both in governmental and opposition news non-governmental respondents performed better. Complementing the findings above, students allocated to the intervention group were better in the discernment of opposition news. See Appendix Figure 1-4.

Based on the analysis, gender, age, elaborateness, formulation (conditional-mode, imperative sentence) and style (loving, dutiful) of the letter were within the ten most important features when classifying the performance of the students on the discernment task. (see further details in Conclusion).

**Conclusion**

As a result of the analysis, it turned out that there might be some interesting insights considering the role of gender, age, religiousness, intervention group membership, elaborateness, formulation, and style of the letter, therefore, as an improvement on the current analysis, we plan to examine those further in the future.

1. Brownlee, J. XGBoost With Python: Gradient boosted trees with XGBoost and scikit-learn. Machine Learning Mastery (2016).

**Appendix**

**Table S3. The proportion of respondents belonging to the categories of disc_pro_gov_binary**

| disc_pro_gov_binary | Proportion of participants |
| --- | --- |
| 0 | 52,2% |
| 1 | 47,8% |

**Table S4. The proportion of respondents belonging to the categories of disc_pro_opp_binary**

| disc_pro_opp_binary | Proportion of participants |
| --- | --- |
| 0 | 57,7% |
| 1 | 42,3% |

XGBoost classification model 1.

Outcome: disc_pro_opp_binary

The following input variables were used:

- Condition (categorical variable: referring to the intervention- or control groups)
- Gender (categorical variable: male, female)
- Age (continuous variable: ranging from 18 to 53)
- Religiousness (dummy variable: religious or non-religious)
- Strategy (number of strategies observed in the letter)
- Seriousness/Elaboration (categorical variable: bullshit, elaborated, mis-elaborated)
- Autonomy (dummy variable: indicating whether there were significant words of autonomy involved in the letter)
- Conditional (dummy variable: indicating whether there were conditional tenses observed in the letter)
- Imperative (dummy variable: indicating whether there were imperative sentences observed in the letter)
- Loving (dummy variable: indicating whether the style of the letter was loving or not)
- Derogatory (dummy variable: indicating whether the style of the letter was derogatory or not)
- First generation (dummy variable: indicating whether the respondent is a first-generation student or not)
- Pro-governmental (dummy variable: indicating whether the respondent is pro-governmental or non-governmental)

XGBoost classification model 2.

Outcome: disc_pro_gov_binary

The following input variables were used:

- Condition (categorical variable: referring to the intervention- or control groups)
- Gender (categorical variable: male, female)
- Age (continuous variable: ranging from 18 to 53)
- Religiousness (dummy variable: religious or non-religious)
- Strategy (number of strategies observed in the letter)
- Seriousness/Elaboration (categorical variable: bullshit, elaborated, mis-elaborated)
- Autonomy (dummy variable: indicating whether there were significant words of autonomy involved in the letter)
- Conditional (dummy variable: indicating whether there were conditional tenses observed in the letter)
- Imperative (dummy variable: indicating whether there were imperative sentences observed in the letter)
- Loving (dummy variable: indicating whether the style of the letter was loving or not)
- Derogatory (dummy variable: indicating whether the style of the letter was derogatory or not)
- First generation (dummy variable: indicating whether the respondent is a first-generation student or not)
- Pro-governmental (dummy variable: indicating whether the respondent is pro-governmental or non-governmental)

**Figure S4. Feature importance.**


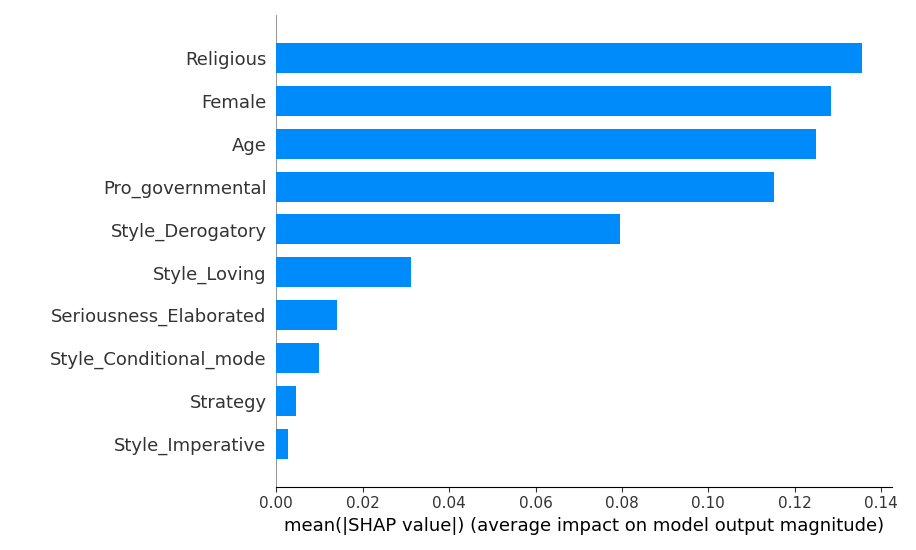


**Figure S5. SHAP values beeswarm plot.**


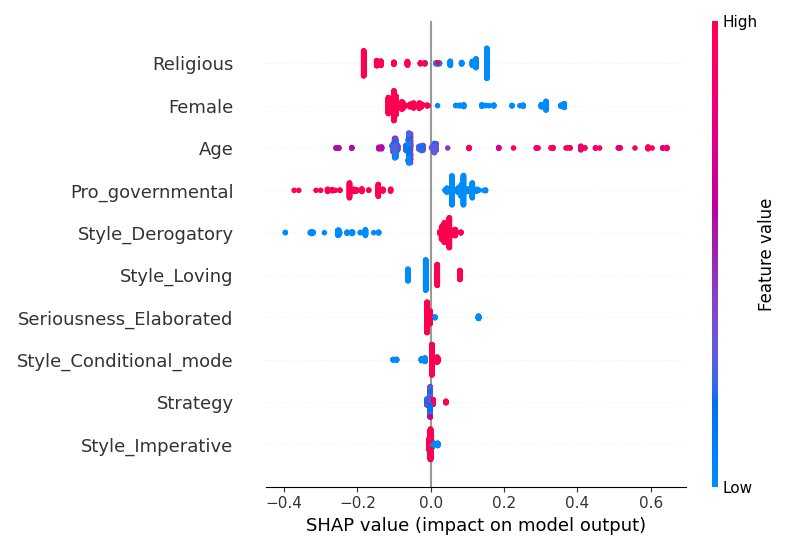

Supplement: Supplementary file 1 — Supplementary Information. [file 41598_2024_61232_MOESM1_ESM.docx]
